# Full genome characterization and phylogenetic analysis of hepatitis B virus in gibbons and a caretaker in Central Kalimantan, Indonesia

**DOI:** 10.1007/s00705-014-2323-9

**Published:** 2015-01-07

**Authors:** Takako Utsumi, Rury Mega Wahyuni, Maria Inge Lusida, Yoshihiko Yano, Nur Purba Priambada, Mochamad Amin, Priyo Budi Purwono, Anittaqwa Istimagfiroh, Soetjipto, Aurélien Brulé, Hak Hotta, Yoshitake Hayashi

**Affiliations:** 1 Indonesia-Japan Collaborative Research Center for Emerging and Re-emerging Infectious Diseases, Institute of Tropical Disease, Airlangga University, Surabaya, Indonesia; 2 Kalaweit Gibbon Conservation Center and Sanctuary, Central Kalimantan, Indonesia; 3 Center for Infectious Diseases, Kobe University Graduate School of Medicine, 7-5-1 Kusunoki-cho, Chuo-ku, Kobe, Hyogo 650-0017 Japan

## Abstract

Hepatitis B virus (HBV) from gibbons was characterized, and the possibility of horizontal transmission between gibbons and humans was examined in a gibbon rehabilitation center in Central Kalimantan, Indonesia. Ten gibbons that were positive for the hepatitis B surface antigen (HBsAg) on arrival and 13 caretakers for those gibbons were included in this study. The duration of stay at the rehabilitation center ranged from 1 to 10 years. Serological and molecular analyses were performed. Six gibbons were positive for HBsAg, whereas HBV DNA was detected in all ten of the gibbons sampled. On the other hand, HBsAg was detected in only 1 of the 13 caretakers. HBV samples from seven gibbons and from the one infected human were chosen for complete genome sequencing. A phylogenetic analysis revealed that the cluster of gibbon strains in this study was distinct from strains previously reported from other countries. In the pre-S1 region, we found a unique amino acid residue substitution (P89K), three insertions between T87 and L88 in the genomes of three gibbons, and a 33-nucleotide deletion at the start of pre-S1 that is common in non-human primates. The caretaker sample was identified as HBV subgenotype B3, the most common type in Indonesia. For the complete HBV sequences, the similarity between gibbons in this study and other non-human primate and human HBV isolates was 90–91.9 % and 85.5–89.6 %, respectively. In conclusion, the gibbon HBV genotype was influenced by geographic location and species. To the best of our knowledge, this is the first report characterizing the HBV genes and genomes of indigenous gibbons in Indonesia.

## Introduction

Hepatitis B virus (HBV) is present worldwide, and chronic HBV infection frequently results in cirrhosis and hepatocellular carcinoma (HCC). HBV is the smallest mammalian DNA virus, with a genome size of approximately 3,200 nucleotides that contains four open reading frames for the P, C, S, and X genes. HBV variants have been classified into at least nine genotypes (A through H and J) for humans [1, 2], and genomic differences distinguish strains infecting humans from those infecting non-human primates, including chimpanzees, orangutans, gorillas, and gibbons [3, 4]. Human HBV genotypes have a distinct geographical distribution and differ in the clinical manifestations they induce [5, 6], but it is unclear whether this is also true of non-human HBV genotypes [4].

HBV can be found in non-human primates of the families *Hominidae* (chimpanzee, gorilla, and orangutan) and *Hylobatidae* (gibbon), which are distributed across Africa (chimpanzee and gorilla) and Southeast Asia (orangutan and gibbon), which are endemic areas of human HBV [7–9]. The prevalence of asymptomatic HBV carriers is 23–33 % in gibbons and 15 % in orangutans [4]. The genome organization of non-human primate HBVs is nearly identical to that of human HBVs [4]. Because of this close similarity, cross-transmission of HBV between species has been speculated to occur. There have been many studies on the cross-transmission of human HBVs to non-human primates [10, 11], but a cross-transmission of HBVs from non-human primates to humans has not yet been reported [3, 4, 11].

A recent study has shown that a novel HBV strain (genotype “J”, HBV/J) discovered in an elderly Japanese male patient with HCC who was involved in military action in Borneo (Kalimantan) during World War II is phylogenetically intermediate to human and gibbon/orangutan strains [12]. Although it is not certain, HBV/J in gibbon/orangutan or human inhabitants is likely to have originated in Borneo (Kalimantan).

We have investigated HBV infection of gibbons and possible horizontal transmission between gibbons and humans and confirmed the presence of HBV/J in Kalimantan, Indonesia.

## Materials and methods

### Study population

A total of 142 captive gibbons were kept at Kalaweit Gibbon Conservation Center and Sanctuary (Kalaweit), Central Kalimantan, Indonesia, in 2012. Ten out of 15 gibbons that tested positive for hepatitis B surface antigen (HBsAg) in the screening upon arrival at Kalaweit were randomly selected for this study. All of them were born in the wild. The population consisted of six males and four females ranging from 3 to 17 years old and of two species, *Hylobates albibarbis* (Bornean white-bearded gibbons, n = 7) and *Hylobates mulleri* (Müller’s Bornean gibbons, n = 3), originally found in Kalimantan. The Kalaweit animal caretakers (n = 13; mean age, 28 years) included in this study consisted of nine males and four females.

Demographic data for the gibbons and humans were collected from each animal caretaker and veterinary coordinator in Kalaweit. Written informed consent was obtained from all caretakers, and a research permit was obtained from the Ministry of Forestry in Indonesia. The study protocol was reviewed and approved by the Ethics Committees of Kobe University in Japan and Veterinary Medicine of Airlangga University in Indonesia.

### Sample collection

Gibbon blood samples were obtained by venepuncture during a brief period of anesthesia with ketamine, part of the routine health-care programme, in May (5 samples) and November (5 samples) 2012. Human blood was taken from animal caretakers in October 2013. In total, serum samples were collected from 10 gibbons and 13 animal caretakers.

### Serological test

All serum samples were tested for HBsAg by reverse passive hemagglutination (R-PHA) (Mycell II HBsAg; Institute of Immunology, Tokyo, Japan), for antibodies to HBsAg (anti-HBs) by passive hemagglutination (PHA) (Mycell II anti-HBs; Institute of Immunology), and for antibodies to hepatitis B core antigen (anti-HBc) by PHA (Mycell anti-rHBc; Institute of Immunology) according to the manufacturer’s instructions. All sera were confirmed to be HBsAg positive using an enzyme-linked immunosorbent assay (ELISA) (Hepalisa HBsAg PT INDEC DIAGNOSTICS, Jakarta, Indonesia). In addition, serological markers related to active hepatitis, alanine aminotransferase (ALT), aspartate aminotransferase (AST), and α-fetoproteins (AFP), were examined.

### Detection of HBV DNA and viral load in gibbons

The sera were stored at −20 °C until the assay was carried out. Sample DNA was extracted from 200 µL of serum using a DNA extraction kit (QIAamp DNA Blood Mini Kit; QIAGEN, Tokyo, Japan). The presence of HBV DNA and the viral load were assessed by real-time PCR using an ABI PRISM 7300 Analyzer (Applied Biosystems, Foster City, CA). HBV was amplified using a previously described primer and probe set [13].

### Nucleotide sequence analysis

After being assayed for their HBV serological status, all serum samples were subjected to a HBV genetic analysis. The complete HBV genome sequences were determined by a method reported previously [14]. In brief, the complete genome of HBV was first amplified as two overlapping fragments, a 3,200-bp amplicon (fragment A) and a 462-bp amplicon (fragment B) covering the remaining region. Fragment A was then subjected to nested PCR to amplify 11 overlapping fragments. The amplified fragments were sequenced directly using a Big Dye Deoxy Termintor Cycle Sequencing Kit with an ABI PRISM 310 Genetic Analyzer (Applied Biosystems, Foster City, CA).

### Phylogenetic analysis

Reference sequences were retrieved from the DDBJ, EMBL, and GenBank databases. Sequences were aligned using the Clustal X multiple sequence alignment program. Phylogenetic trees were constructed using the neighbor-joining method, and bootstrap resampling was performed 1,000 times. These analyses were carried out using the Molecular Evolutionary Genetics Analysis (MEGA) program. Subgenotypes were assigned as described previously [3, 15, 16].

### Nucleotide sequence accession numbers

The nucleotide sequence data reported in this paper were deposited in the DDBJ, EMBL, and GenBank databases under accession nos. AB823656 through AB823662 for the gibbon HBV sequences and AB976562 for the caretaker sequence.

## Results

### Seroprevalence of HBV in gibbons and humans

To determine the prevalence of current HBV infection in gibbons and animal caretakers in Kalaweit, 23 serum samples (10 gibbons and 13 caretakers) were tested for the presence of HBsAg, anti-HBs, and anti-HBc. A total of six gibbons were chronic carriers, as defined by the presence of HBV DNA and HBsAg in the absence of antibody to protein S, and the remaining four gibbons showed previous infection. All 10 gibbons were positive for at least one marker of HBV infection (Table 1), and one of the 13 animal caretakers (7.7 %) was positive for HBsAg. One gibbon was negative for anti-HBc in the presence of HBsAg (Table 1). ALT, AST, and AFP levels were within the normal range for all gibbons. Anti-HBs antibodies were found in 10 of the 13 caretakers, and 8 of those 10 were positive for anti-HBc.

**Table 1:** Demographic data, HBV serological markers, and viral load in gibbons

ID	Age (yrs)	Sex	Species	Duration in the center (yrs)	HBsAg	Anti-HBs	Anti-HBc	HBV viral load (log copies/mL)
GB1	15	M	*Hylobates albibarbis*	5	+	−	+	7.7
GB2	15	M	*Hylobates mulleri*	6	−	−	+	4.4
GB3	10	F	*Hylobates albibarbis*	9	−	+	+	3.9
GB4	16	F	*Hylobates mulleri*	8	−	−	+	4.1
GB5	17	M	*Hylobates mulleri*	10	+	−	+	6.5
GB6	8	M	*Hylobates albibarbis*	1	+	−	+	7.0
GB7	8	F	*Hylobates albibarbis*	2	+	−	+	7.5
GB8	4	M	*Hylobates albibarbis*	3	−	+	+	3.9
GB9	3	F	*Hylobates albibarbis*	1	+	−	+	6.4
GB10	4	M	*Hylobates albibarbis*	3	+	−	−	7.1

### HBV DNA detection in gibbons and caretakers

HBV DNA was detected in all samples regardless of HBsAg status. The HBV viral load was higher in HBsAg-positive samples (mean, 7.0; 6.4–7.7 log copies/mL) than in HBsAg-negative samples (mean, 4.1; 3.0–4.4 log copies/mL) (Table 1). Four HBsAg-negative gibbons had occult HBV-like infection (Table 1). HBV DNA was also detected in one caretaker with HBsAg.

### Gibbon and human HBV nucleotide and amino acid sequences

The complete HBV genomes from seven gibbons comprised 3,182–3,191 nucleotides and showed a genetic organization similar to that of the human viruses. We compared the HBV sequences of all seven gibbons and the human obtained in this study with representative sequences in GenBank, including nine human HBV genotypes, orangutan, gibbon, chimpanzee, and gorilla HBV sequences. The nucleotide sequence of the GB1 isolate (accession no. AB823656) in this study showed similarities ranging from 85.5 to 91.9 % with known complete HBV genome sequences. The highest degree of similarity was found for GB1 and the HBV genomes of viruses isolated from Thai gibbon (accession no. EU155829), orangutan (accession no. AF193863), and chimpanzee (accession no. AF222323) (91.9 %, 91.2 %, and 90.6 % similarity, respectively). Interestingly, the GB1 isolate had higher similarity, not only with primate HBV but also with the human HBV genotypes C and J (92.9 % and 94.1 %), in the pre-C/C region than in the full-length sequence.

### Complete HBV genome sequences

We compared the HBV sequences from the seven gibbons with each of those from other primates, as well as those of human HBV genotypes A through H and J. We observed 91.6–99.1 % sequence identity for pairwise comparisons with the Kalaweit gibbon HBV sequences. The gibbon HBV sequences from this study showed 90.7–92.3 % sequence identity to those from orangutan origin previously found in Kalimantan [15].

### Pre-S/S gene

**Fig. 1:**
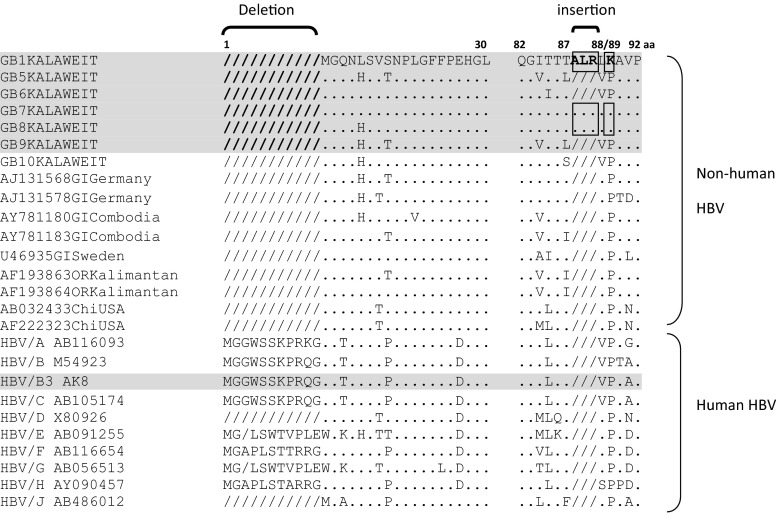
Amino acid sequence alignment of the pre-S1 region of seven gibbon HBV strains (G1, G5, G6, G7, G8, G9, and G10) and an isolate from a human caretaker with HBV sequences from five gibbons, two orangutans, and two chimpanzees, as well as nine human HBV sequences from databases. The sequence of the G1 isolate is indicated at the top. Dots represent amino acids shared by G1, and a dash indicates the deletion of an amino acid

We aligned the nucleotide sequences of the pre-S/S region to identify nucleotide and amino acid differences between the HBV isolates from gibbons and the human. Moreover, we compared the gibbon and human HBV sequences with those of previously reported HBV strains. In comparison to the pre-S/S genes of isolates from humans and other species, the isolates from the seven gibbons in this study had a deletion of 33 nucleotides, representing 11 codons at the 5′ end of the pre-S1 region, consistent with previous results [2, 6, 11] (Fig. 1). For the GB1, GB7, and GB8 HBV isolates, we discovered insertions of Ala (A), Leu (L), and Arg (R) between Thr^87^ (T) and L^88^, and a substitution of Lys^89^ (K) (Fig. 1). On closer investigation, the only gibbon-specific HBV amino acids we found in the pre-S1 region were His^57^ (H), L^88^, Ser^88^ (S), and H^100^ (Fig. 1). Those in the pre-S2 region included L^10^, Phe^20^ (F), Tyr^21^ (Y), and L^35^, and those in the S region included Val^4^ (V), Met^28^ (M), Glu^44^ (Z), L^53^, A^114^, M^118^, L^154^, T^210^, S^216^, Trp^221^ (W), and Ile^222^ (I). The amino acids in the *a* determinant region were highly conserved among gibbons, with the exception of an A^131^ observed in the GB9 isolate.

### Pre-C/C gene

We observed less sequence divergence among isolates in the pre-C/C region than in the pre-S/S gene. Three gibbon HBV sequences had a G-to-T mutation, and one had a G-to-A mutation at position 1896; two had a T-to-C mutation at position 1753. The gibbon HBV sequences did not have the double mutations at positions 1762 and 1764 that are commonly observed in humans. The core protein amino acids were highly conserved among the gibbon HBV sequences from this study. The only gibbon HBV amino acid substitutions were Leu^11^ (L) in the pre-C region and Ser^67^ (S) and Pro^127^ (P) in the C region.

### Phylogenetic analysis of gibbon and human HBV

**Fig. 2:**
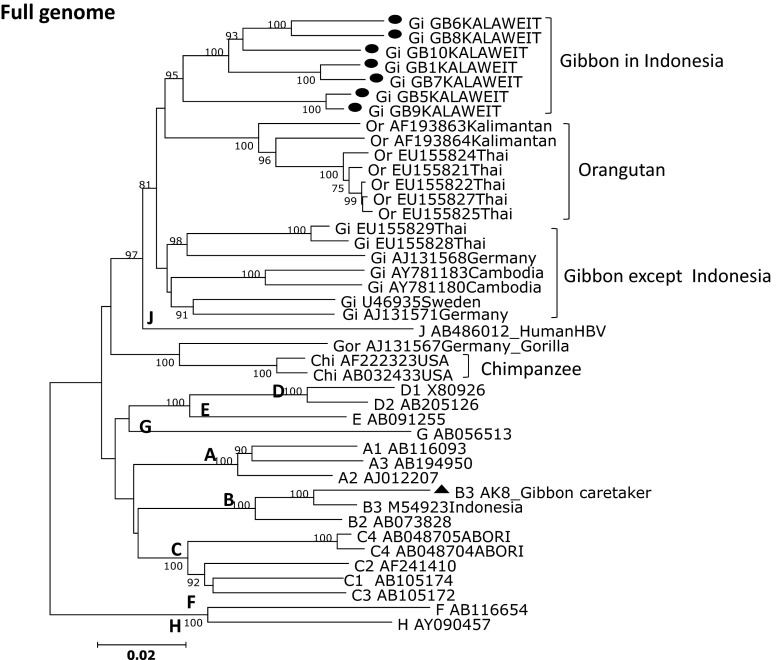
Phylogenetic tree based on complete genome sequences of HBV strains isolated from seven gibbons and a human in Kalimantan along with 34 reference strains. The numbers at the nodes indicate the bootstrap reliability. The lengths of the horizontal bars indicate the number of nucleotide substitutions per site. The country of origin and accession numbers are provided for isolates from the databases

**Fig. 3:**
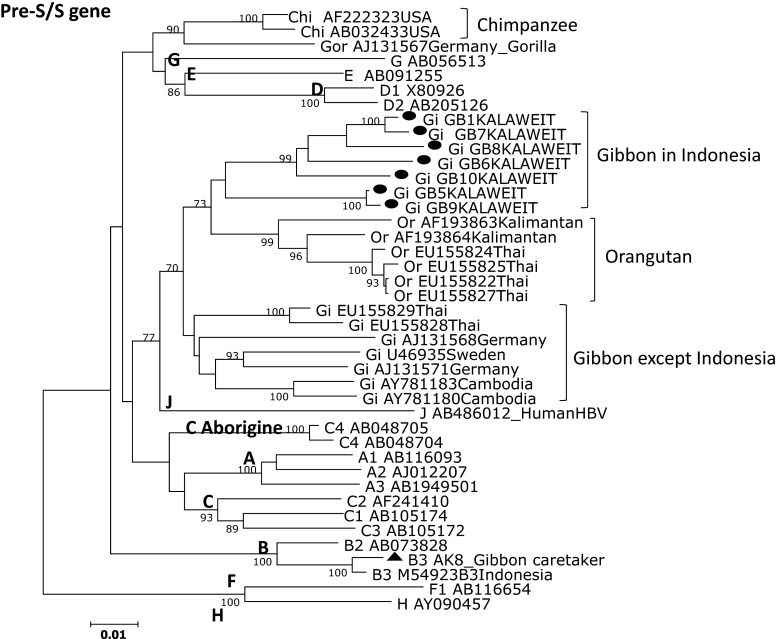
Phylogenetic tree based on pre-S/S gene sequences of HBV strains isolated from seven gibbons and a human in Kalimantan along with 34 reference strains. The numbers at the nodes indicate the bootstrap reliability. The lengths of the horizontal bars indicate the number of nucleotide substitutions per site. The country of origin and accession numbers are provided for isolates from the databases

**Fig. 4:**
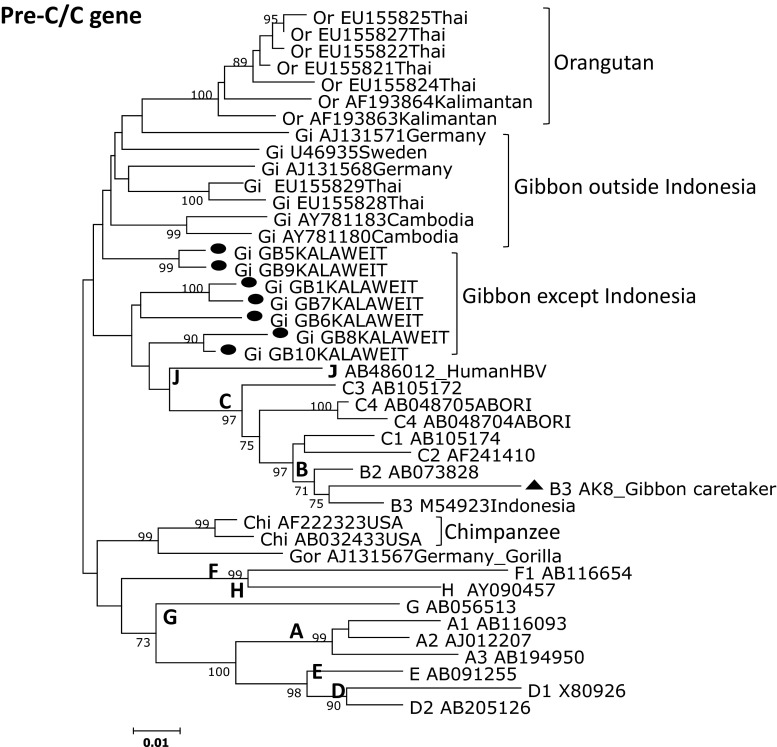
Phylogenetic tree based on pre-C/C gene sequences of HBV strains isolated from seven gibbons and a human in Kalimantan along with 34 reference strains. The numbers at the nodes indicate the bootstrap reliability. The lengths of the horizontal bars indicate the number of nucleotide substitutions per site. The country of origin and accession numbers are provided for isolates from the databases

Of the 11 serum samples (10 gibbons and one human) obtained in Kalaweit, we successfully determined the complete HBV genome sequences for eight samples. We constructed phylogenetic trees of the complete nucleotide sequence, the pre-S/S region, and the pre-C/C region (Fig. 2–4). The phylogenetic analysis included the eight gibbon HBV strains and 52 HBV strains from DDBJ, EMBL, and GenBank. The gibbon HBV strains were classified as Kalaweit gibbon HBV, and the HBV from the human caretaker as human HBV genotype B3 (HBV/B3).

The Kalaweit gibbon HBV strains formed a distinct cluster, separate from the previously reported gibbon HBV strains from Thailand, Cambodia, and Germany [3, 16–18]. A high bootstrap value (95 %) supported the clustering of the gibbon HBV sequences from Kalimantan in the phylogenetic analysis of the complete genome. The subgenotype of the caretaker, HBV B3, was the most common type observed in Indonesia. Furthermore, the gibbon HBV isolates (GB1, GB5, GB6, GB7, GB8, GB9, and GB10) were more distantly related to the HBV sequences from orangutans previously found in Kalimantan (accession no. AF193863, AF193864) than they were to gibbon HBV strains from other regions, except Thailand. Although one gibbon caretaker was found to be a chronic HBV carrier in this study, we did not find any evidence for zoonotic disease transmission. The data for the complete genome and pre-S/S gene support separate clusters for the human HBV isolates and the non-human primates (Figs. 2 and 3). The Kalaweit gibbon HBV strains showed a closer relationship to other HBV genotypes in the pre-C/C gene (Fig. 4).

## Discussion

We performed a complete genome sequence analysis and found that HBV in *Hylobates albibarbis* and *Hylobates mulleri* originally found in Kalimantan clustered into a single group (Fig. 2). To the best of our knowledge, this is the first report of HBV genotypes for indigenous gibbons in Indonesia. On the other hand, the HBV subgenotype of a gibbon caretaker in Kalaweit was B3, the most common type observed in Indonesia [19, 20], supporting previous work showing that human HBV genotypes have a distinct geographical distribution. HBV cross-transmission between gibbons and humans was not supported by our phylogenetic analysis. The Kalaweit gibbon HBV strains had variation in the pre-S region, including a unique amino acid residue substitution (P89 K), three insertions (found in three gibbons), and a 33-nucleotide deletion that is common in non-human primates (Fig. 1). All ten gibbons in this study were HBsAg positive as determined by routine screening of new gibbons arriving at Kalaweit; they were already infected with gibbon HBV before encountering caretakers. Immunization was recommended for the protection of Kalaweit caretakers, and approximately 40 % of caretakers were immunized with the hepatitis B vaccine. In addition, none were susceptible.

Previous reports indicate that the origin of HBV infection in humans and other primates is unresolved [3, 10]. We suspect that the new HBV/J recently found in a Japanese HCC patient infected individuals in Kalimantan, because it is more closely related to human than to non-human HBV strains [12], with a 33-nucleotide deletion in the pre-S1 region unique to non-human primate HBV. This discovery is particularly important for the inhabitants of Kalimantan, because the HBV/J strain is associated with HCC, an advanced stage of chronic hepatitis B. Hence, it is necessary to monitor the spread of HBV/J in Kalimantan among human and non-human primates. We did not find HBV/J in this study. Interestingly, the HBV/J strain was more closely related to the gibbon strains in this study in the core region than in the complete genome and S region, both of which were found to be distinct. We also did not find the HBV/J genotype in our studies of HBV among asymptomatic carriers and chronic HBV patients in East Kalimantan (Utsumi et al., unpublished data), suggesting that this strain is not widespread in Indonesia. This also suggests that cross-infection between humans and non-human primates is extremely rare. Indonesia is an endemic area for HBV infection. Several studies on orangutan genetics have been carried out in Kalimantan [15, 21]. There are no reports of HBV transmission between human and non-human primates in Indonesia. However, the discovery of HBV/J suggests that cross-transmission is possible, and the genome organization of non-human primate HBVs is nearly identical to that of human HBVs. Considering the discovery of HBV/J in Kalimantan, it is necessary to accumulate additional molecular epidemiological data on HBV infection in human and non-human primates inland of Kalimantan.

The prevalence of HBV carriers in primates is approximately 20–30 % or more [4, 22]. Because we could only obtain ten serum samples from gibbons at Kalaweit, the prevalence of HBV is not clear. Of the ten gibbons, six were still positive for HBsAg since arriving at Kalaweit (the duration of stay ranged from 1 to 10 years) (Table 1) and were considered chronically infected. Interestingly, in the four remaining HBsAg-negative gibbons, we detected HBV DNA; this pattern was consistent with a human HBV infection called occult HBV infection, characterized by the absence of HBsAg and the presence of HBV DNA. Occult HBV infection has not yet been reported in non-human primates. Human occult HBV infection is possibly caused by changes in antigenicity [23]. In this study, we did not observe a specific mutation in the *a* determinant region that could influence antigenicity, so further research is needed to confirm this occult-HBV-infection-like phenomenon. In addition, even though the viral load was low in HBsAg-negative gibbons, it was detectable and sufficient for sequencing; it could be a source of virus spread in the gibbon population. We need to be aware that these infections have been detected in captive animals, which may have been exposed to other HBV-infected hosts during captivity. The C^1753^, A^1896^, and T^1896^ mutations in the core region, which have been associated to advanced liver disease [24], were found in three gibbons. The precise clinical course of gibbons infected with HBV is not fully understood. In this study, HBsAg-positive gibbons had higher HBV-DNA levels compared with HBsAg-negative ones, and anti-HBs was detected only in gibbons that were HBsAg negative and had low HBV-DNA levels (Table 1). This suggests that HBs seroconversion occurs after HBsAg seroclearance. Due to the similarity between the genetic organization of human and non-human primate HBV, the clinical manifestations may be similar to hepatitis induced by human HBV [12]. This requires further investigation.

In conclusion, a phylogenetic analysis of complete genome sequences revealed that gibbon HBV strains in Central Kalimantan formed a distinct cluster, separate from hepadnaviruses of other hosts. A unique amino acid residue (P89 K), three insertions, and other point variations in the pre-S region contribute to this distinct HBV genotype, and the geographic location and host species influenced the gibbon HBV genotype [25]. To the best of our knowledge, this is the first report characterizing the HBV genes and genomes of indigenous gibbons in Indonesia.
